# Direct methanol synthesis from methane in a plasma-catalyst hybrid system at low temperature using metal oxide-coated glass beads

**DOI:** 10.1038/s41598-018-28170-x

**Published:** 2018-07-02

**Authors:** Heesoo Lee, Do Heui Kim

**Affiliations:** School of Chemical and Biological Engineering, Institute of Chemical Processes, Seoul National University, 1 Gwanak-ro, Gwanak-gu Seoul, 08826 Republic of Korea

## Abstract

A plasma-catalyst hybrid system was used to synthesize methanol directly from methane. A dielectric barrier discharge (DBD) plasma combined with the catalyst was introduced in order to overcome the difficulties of catalyst-only batch reactions such as high reaction pressure and separation of liquid product. Of the transition metal oxides, Mn_2_O_3_-coated glass bead showed the highest methanol yield of about 12.3% in the plasma-catalyst hybrid system. The reaction temperature was maintained below 100 °C due to the low plasma input power (from 1.3 kJ/L to 4.5 kJ/L). Furthermore, the reactivity of the catalyst was maintained for 10 hr without changing the selectivity. The results indicated that the plasma-induced OH radical might be produced on the Mn_2_O_3_ catalyst, which led to methanol synthesis.

## Introduction

Methane is a main component of natural gas, which has large reserves spread all over the world^1^. Therefore, methane conversion to value-added products has drawn much attention because of scientific and industrial significance^2^. Currently, the production of methanol from natural gas should go through the route of synthesis gas, which is considered to be energetically inefficient. Thus, direct methanol synthesis from methane has been steadily investigated by many researchers^3–5^. However, direct methanol synthesis through catalytic reactions has limitations given that a large quantity of thermal energy is required to selectively activate the C-H bonds. Unfortunately, introducing thermal energy results in unwanted oxidation of methane into CO or CO_2_. To prevent this, a batch reaction has been carried out under a high pressure using liquid-phase reactants with expensive oxidants such as H_2_O_2_^6–10^. Though this could enhance the methanol selectivity, the methanol yield remains low (<10%), and separation and regeneration processes are needed^11^.

This study introduces plasma to overcome such difficulties. Plasma can activate methane to produce a methyl radical at lower temperatures^12^. The plasma can be divided into thermal plasma and non-thermal plasma by the degree of thermal equilibrium^13^. The temperatures of electron, ion, and radical become identical in the thermal plasma condition. On the contrary, while electron temperature is high, the temperature of bulk gas can be maintained at low temperature in the non-thermal plasma condition^14^. This is because the mass of electron that is accelerated by plasma is very light, which leads to increase of the gas temperature by only a few degrees^15,16^. Accordingly, partially activated gas including ion and radical can be generated in a simple reactor configuration^17^. At this point, the reaction atmosphere can remain isothermal condition. Because of this circumstance, structural variation of the catalyst can be hardly created in non-thermal plasma condition using a simple reactor configuration^18^. There are diverse plasma sources such as corona, microwave, arc, spark, radio frequency, and dielectric barrier discharge (DBD)^19^. Among these various plasma sources, dielectric barrier discharge (DBD) plasma has been generally applied by former researchers because it can be formed at atmospheric pressure where other non-thermal plasmas are hardly generated^20–24^. Therefore, DBD plasma was selected as a plasma source in this study. The system was designed with the plasma activated methane and catalyst that play a role in improving the methanol selectivity in a continuous packed bed reactor which does not require separation and regeneration processes.

## Results and Discussion

### Plasma only reaction

First, the experiment was conducted under plasma only condition as the baseline for the plasma-catalyst hybrid reaction. In the plasma only reaction, the methane conversion and methanol selectivity were measured in the range of plasma input voltage from 6.5 kV_P-P_ to 8.5 kV_P-P_ at intervals of 0.5 kV_P-P_ at low temperature. Due to the plasma property, methane was able to activate and oxidize without adding any thermal energy. Aside from CO, CO_2_, and methanol, oxygenated products were hardly detected. Among the products, CO was the most abundant during the reaction. The plasma power calculated from V-Q Lissajous figure changed from 2.3 W to 7.2 W (from 1.4 kJ/L to 4.3 kJ/L), depending on the input voltage that led to variations in the reaction temperature from 61 °C to 108 °C. As the input voltage increased, the methanol selectivity decreased since the methanol was more oxidized to the products such as CO and CO_2_. However, the methane conversion also increased due to an increase in the plasma power. Consequently, the overall methanol yield increased from 5.1% to 8.7% while the input voltage varied from 6.5 kV_P-P_ to 8.5 kV_P-P_ (Table 1). The methanol yield was confirmed to steadily increase although the methanol selectivity decreased as the input voltage went up in this reaction condition. According to Zhou *et al*., the DBD plasma can provide electrons with high energy enough to dissociate CH_4_ and O_2_^25^. Under the plasma condition, the oxygen radical, methyl radical, and hydrogen radical can be produced. In addition, excited oxygen atom can react with CH_4_ to produce CH_3_ and OH. Possible reaction pathways for methanol synthesis under plasma condition are described in Table 2.

**Table 1:** Direct methanol synthesis from methane under plasma only condition.

Input voltage (kV_P-P_)	Specific input energy (kJ/L)	Selectivity(%)			CH_4_ conversion(%)	CH_3_OH yield(%)
		CO	CO_2_	CH_3_OH		
6.5	1.4	47.5	14.3	33.0	15.4	5.1
7.0	2.0	47.5	15.1	33.8	19.4	6.5
7.5	2.9	49.6	15.0	32.6	23.3	7.6
8.0	3.4	50.4	14.6	32.1	25.7	8.2
8.5	4.3	51.7	15.7	29.7	29.1	8.7

**Table 2:** Possible reaction pathways for direct methanol synthesis from methane under a plasma condition.

Reaction	Rate coefficient (cm^3^·molecule^−1^·s^−1^)		Reference
Radical formation	${{\rm{CH}}}_{3}+{\rm{O}}\to {{\rm{CH}}}_{2}{\rm{O}}+{\rm{H}}$	1.3×10^−10^	^34^
	${{\rm{CH}}}_{3}+{{\rm{HO}}}_{2}\to {{\rm{CH}}}_{3}{\rm{O}}+{\rm{OH}}$	3.3 × 10^−11^	^34^
	${{\rm{CH}}}_{3}{{\rm{O}}}_{2}+{\rm{H}}\to {{\rm{CH}}}_{3}{\rm{O}}+{\rm{OH}}$	1.6 × 10^−10^	^34^
	${{\rm{CH}}}_{3}{{\rm{O}}}_{2}+{\rm{O}}\to {{\rm{CH}}}_{3}{\rm{O}}+{{\rm{O}}}_{2}$	6.0 × 10^−11^	^34^
	${{\rm{CH}}}_{3}{{\rm{O}}}_{2}+{{\rm{CH}}}_{3}\to {{\rm{CH}}}_{3}{\rm{O}}+{{\rm{CH}}}_{3}{\rm{O}}$	4.0 × 10^−11^	^34^
	${{\rm{CH}}}_{2}{\rm{O}}+{\rm{H}}\to {{\rm{CH}}}_{3}{\rm{O}}$	1.0 × 10^−10^	^34^
Methanol formation	${{\rm{CH}}}_{2}{\rm{OH}}+{\rm{HCO}}\to {{\rm{CH}}}_{3}{\rm{OH}}+{\rm{CO}}$	2.0 × 10^−10^	^35^
	${{\rm{CH}}}_{3}{{\rm{O}}}_{2}+{\rm{OH}}\to {{\rm{CH}}}_{3}{\rm{OH}}+{{\rm{O}}}_{2}$	1.0 × 10^−10^	^34^
	${{\rm{CH}}}_{3}{\rm{O}}+{{\rm{CH}}}_{3}{\rm{O}}\to {{\rm{CH}}}_{3}{\rm{OH}}+{{\rm{CH}}}_{2}{\rm{O}}$	1.0 × 10^−10^	^34^

### Glass bead with plasma reaction

**Figure 1:**
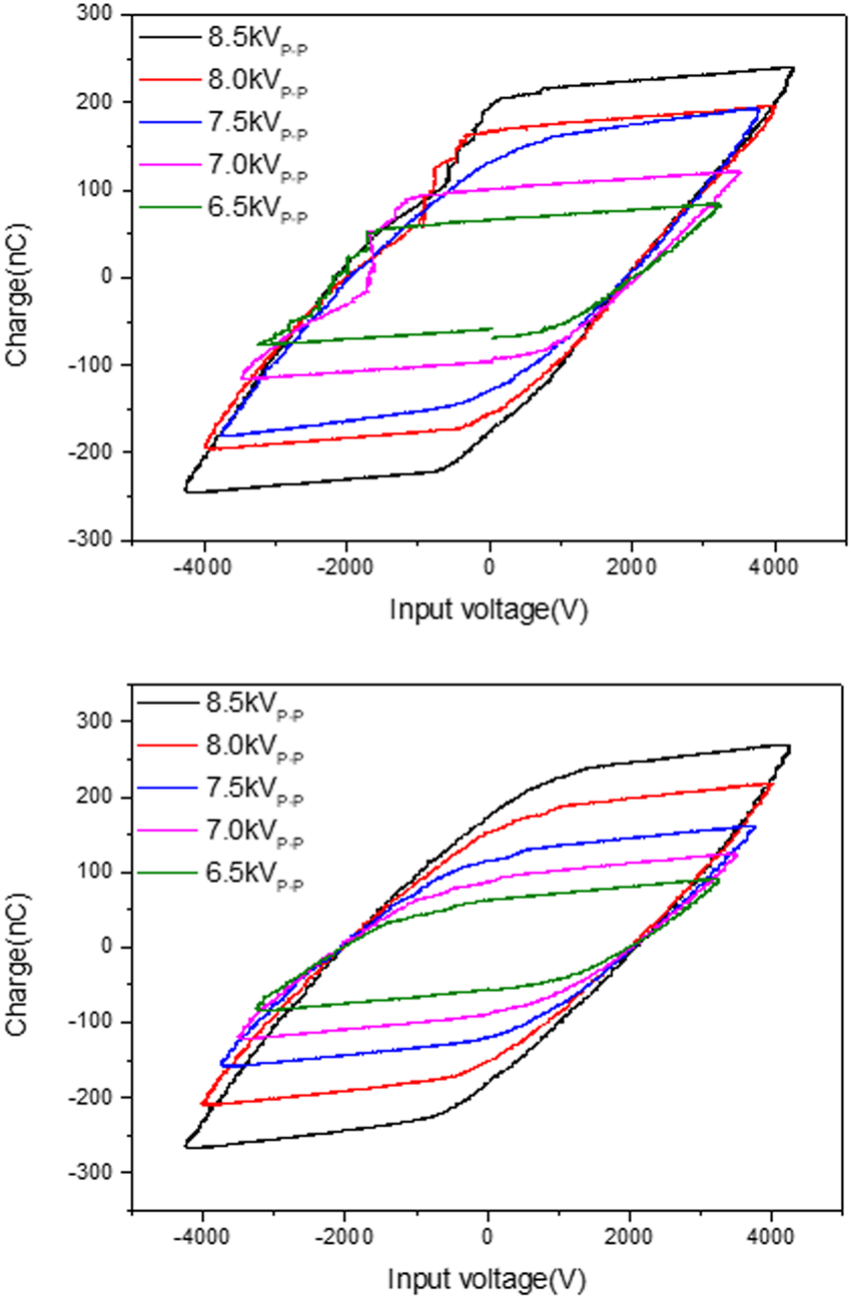
Lissajous figure of plasma only condition (top) and glass bead under plasma condition (bottom).

To improve the methanol productivity, glass beads were loaded into the reactor as a packing material. Before loading, the glass beads were etched using a 5 M NaOH solution to improve the adhesion of the metal oxide. After inserting the glass beads, the plasma configuration became much more stable, as shown in Fig. 1, due to the spherical shape and low dielectric constant of the glass beads. In addition, packing material like glass bead is helpful for expanding the discharge reaction since the streamers or microdischarges tend to propagate along the solid surface^26^. The electric field would be enhanced by loading packing material because of the short distance in the adjacency of contact points. The plasma energy is apt to be consumed by the electron-impact dissociation and ionization reactions by the enhanced electric field. At all plasma conditions from 6.5 kV_P-P_ to 8.5 kV_P-P_ at low temperature, the reaction of the glass beads with plasma resulted in a higher methanol yield than with the plasma only reaction. The methanol yield increased from 6.3% to 9.6% in the presence of the glass beads (Table 3). However, the methanol selectivity decreased as the input voltage increased higher. The methanol yield was confirmed to hardly increase above 7.5 kV_P-P_. Furthermore, the increase rate, which is the division of the methanol yield in the presence of the glass bead by that of plasma only, decreased from 124.4% to 110.8%. The plasma power varied from 2.2 W to 7.6 W (from 1.3 kJ/L to 4.5 kJ/L) according to the input voltage, which was similar to the plasma only reaction. Considering the reactivity and specific input energy, 7.5 kV_P-P_ was selected as the optimum plasma condition.

**Table 3:** Direct methanol synthesis from methane using glass beads with plasma.

Input voltage (kV_P-P_)	Specific input energy (kJ/L)	Selectivity(%)			CH_4_ conversion(%)	CH_3_OH yield(%)
		CO	CO_2_	CH_3_OH		
6.5	1.3	38.9	12.3	44.0	14.3	6.3
7.0	2.0	44.8	12.5	39.1	20.8	8.1
7.5	2.6	48.3	13.9	34.9	26.6	9.3
8.0	3.6	53.2	14.9	29.3	32.7	9.6
8.5	4.5	55.8	16.2	25.5	37.6	9.6

### Metal oxide-coated glass bead (MO/GB) under plasma condition

**Figure 2:**
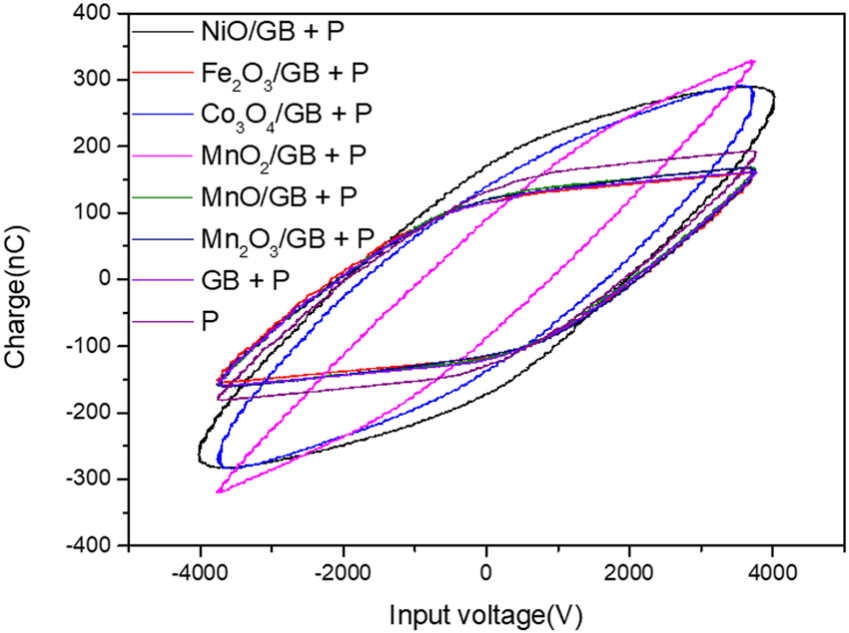
Lissajous figure of metal oxide coated glass bead under plasma condition; GB = Glass bead, P = Plasma.

**Figure 3:**
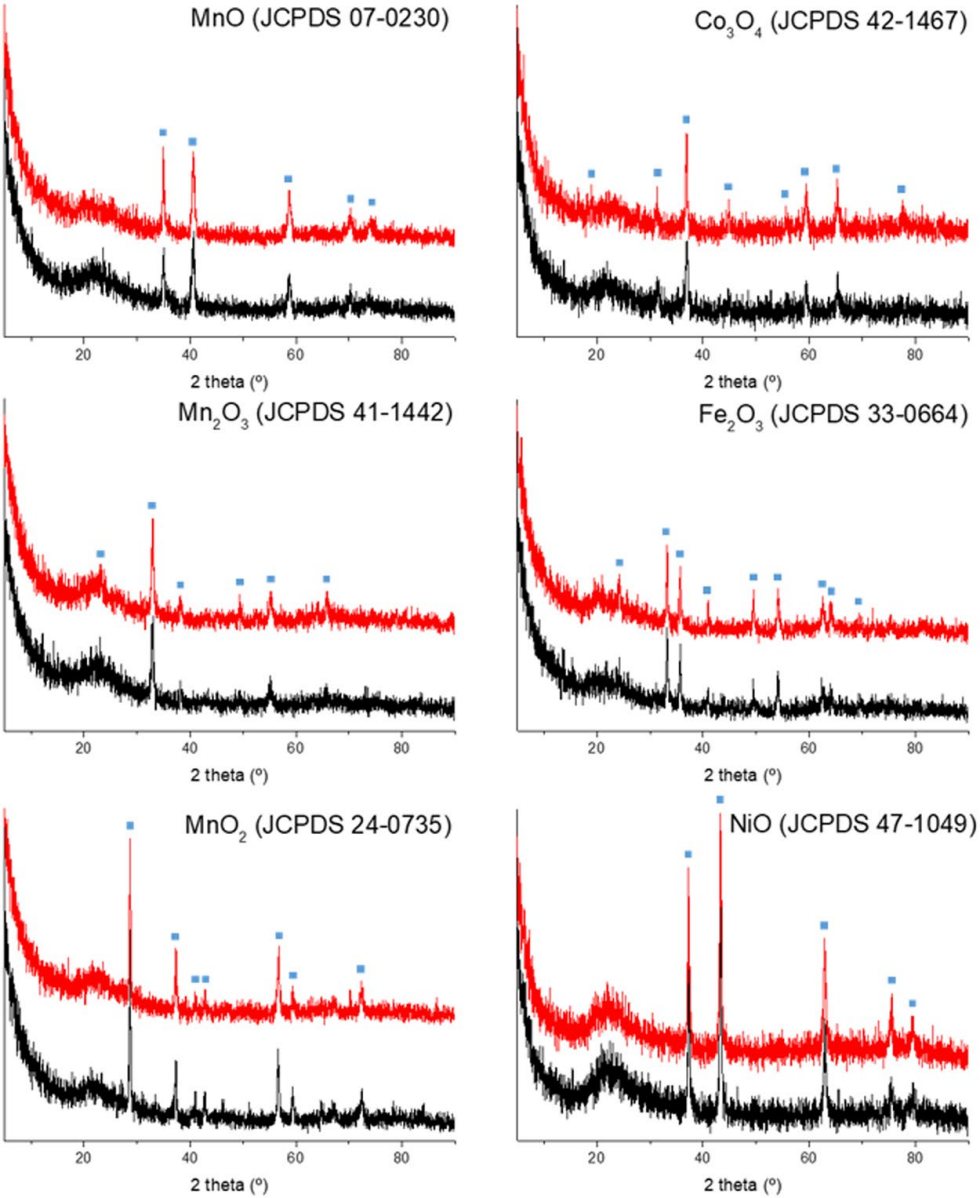
XRD patterns of metal oxide-coated glass bead; Top pattern (red) and bottom pattern (black) of each plot stand for the catalyst before reaction and after reaction, respectively.

The glass beads exhibited a synergistic effect with the plasma to partially oxidize the methane into methanol, so there were further used as the substrate. On the glass beads, some transition metal oxides, such as MnO, Mn_2_O_3_, MnO_2_, Fe_2_O_3_, NiO, and Co_3_O_4_ were coated to improve the activity for methanol synthesis. The surface area of all catalysts was below 1 m^2^/g. As displayed in Table 4, MnO, Mn_2_O_3_, and Fe_2_O_3_ showed a higher activity than the glass beads with plasma. In addition, some catalyst with plasma in the current study demonstrated higher performance than the previous reports which applied plasma only or catalyst only reaction. The catalysts displayed a similar plasma configuration and power as that of the glass beads. Hence, this synergistic effect can be inferred to result not from the plasma alteration but from a catalytic reaction. The ratio of CH_3_OH yield and specific input energy is shown in Table 4. The ratio underlined that the reaction in this study required lower energy to gain same methanol yield than the references. Furthermore, most of the products were CO, CO_2_, and CH_3_OH which were easy to separate. On the other hand, MnO_2_, NiO, and Co_3_O_4_ demonstrated poor activity that was even lower than that of the plasma only condition. In the case of MnO_2_, NiO, and Co_3_O_4_, the plasma configuration became conductive according to the Lissajous figure (Fig. 2). Due to the increased conductivity, the plasma configuration altered significantly, and the plasma was nearly extinguished, which gave rise to a sharp decrease in the methane conversion. Of these, MnO_2_ showed a high methanol selectivity even though it indicated a low methane conversion since plasma hardly formed. After the reaction, all catalysts did not appear to undergo an alteration in structure as shown in Fig. 3. The formation of OH radicals is crucial for direct methanol synthesis from methane^27,28^. According to Guo *et al*., the OH radical can be adsorbed well into the MnO_x_ catalyst^29^. This result suggested that the Mn_2_O_3_ catalyst could increase the possibility of the OH radical to participate in methanol synthesis reaction.

**Table 4:** Methane conversion and product selectivity of the metal oxide-coated glass beads under a plasma condition (7.5 kV_P-P_, 4 kHz).

Samples^a^	Specific input energy (kJ/L)	CH_4_ conversion (%)	Selectivity (%)			CH_3_OH yield (%)	$\frac{{{\bf{CH}}}_{{\bf{3}}}{\bf{OH\; yield}}}{{\bf{Specific\; input\; energy}}}$
			CO	CO_2_	CH_3_OH		
P	2.9	23.3	49.6	15.0	32.6	7.6	2.6
GB B6P	2.6	26.6	48.3	13.9	34.9	9.3	3.6
MnO/GB +GBP	2.7	33.1	48.4	13.7	34.4	11.4	4.2
Mn_2_O_3_/GB +GBP	2.7	30.5	43.8	13.6	40.2	12.3	4.6
MnO_2_/GB +GBP	2.1	10.4	29.8	12.3	53.7	5.6	2.7
Co_3_O_4_/GB +GBP	3.6	13.1	34.4	24.3	31.5	4.1	1.1
Fe_2_O_3_/GB +GBP	2.6	33.2	51.7	14.2	29.3	9.7	3.7
NiO/GB + P	4.5	17.7	47.1	11.6	36.9	6.5	1.4
P^36^^,b^	30.0	41.0	41.5	19.5	19.5	8.0	0.3
Fe-HZSM-5^37^^,c^	—	31.5	—	72.1	10.8	3.4	—

^a^P and GB represent the plasma and glass beads, respectively.

^b^The specific input energy (SEI) is 30 kJ/L without a catalyst at 50 °C.

^c^Temperature: 630 °C, contact time: 2.5 s, oxygen: 15.5 vol%, Si/Fe ratio: 22, without plasma.

**Figure 4:**
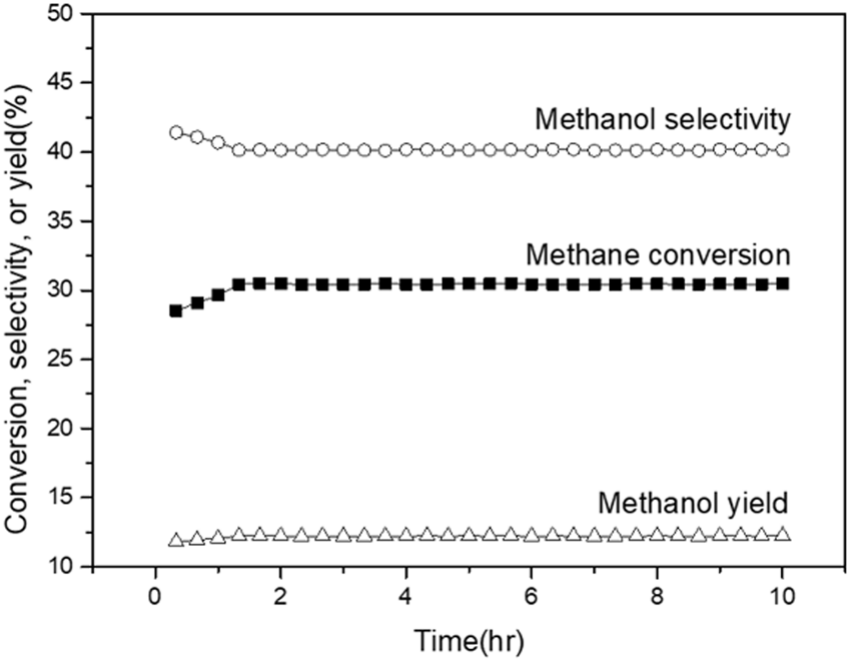
Conversion of methane, selectivity and yield of methanol over Mn_2_O_3_/glass bead as a function of time-on-stream.

The long term activity of the catalyst was explored to confirm the durability of the catalyst and to apply these in a practical application. Figure 4 shows the methane conversion, methanol selectivity, and methanol yield of the Mn_2_O_3_/glass beads showing the highest methanol yield in the plasma-catalyst hybrid system with the time-on-stream (TOS). As shown in Fig. 4, during a reaction for the first one hour, the methane conversion increased, and the methanol selectivity decreased, resulting in a slight increase in the methanol yield. After a reaction for 10 hr, the methanol yield was stabilized at about 12%. This indicated that the plasma-catalyst hybrid system was active and stable for direct methanol synthesis from methane.

## Conclusion

In summary, the plasma-catalyst hybrid system was applied into a direct methanol synthesis from methane. Under the plasma only condition, methane started to become activated to produce methanol below 100 °C. By loading the glass beads, the plasma configuration was stabilized, leading to an increase in the methanol yield. Some transition metal oxides, such as MnO_x_, Fe_2_O_3_, Co_3_O_4_, NiO were coated on the glass beads to improve the activity. Among the catalysts, the Mn_2_O_3_ showed the highest methanol yield of 12.3% when combined with plasma below 100 °C, at an ambient pressure. From the results, it can be implied that the Mn_2_O_3_ catalyst could enhance the possibility of the reaction between OH radical and methane more significantly, which resulted in the highest methanol yield. Furthermore, the stability of the catalyst was maintained for 10 hr, presenting a plasma-catalyst hybrid system that could be a potentially efficient process for direct methanol synthesis from methane.

## Methods

### Reaction system

**Figure 5:**
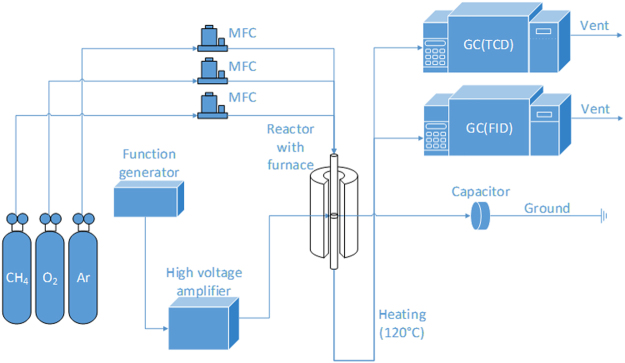
Schematic view of overall reaction system.

Figure 5 indicates the schematic view of overall plasma-catalyst hybrid system. This system was originated and slightly modified from the previous study^30^. Besides, this system is well delineated in the past paper^31^. A 1000:1 high voltage probe (Tektronics P6015A), a current probe (Pearson electronics 6585), and a capacitor (2000 pF) were applied in the system in order to measure voltage (V), current (A), and transferred electric charge (Q), respectively. These signals were sent to a 100 MHz digital oscilloscope (Tektronics DPO 2014). The plasma discharge power was calculated by V-Q Lissajous figure method using the output signal data observed from the digital oscilloscope.

In the middle of the reactor, there was plasma discharge zone. Within the plasma discharge zone, the packing materials such as glass bead and metal oxide-coated glass bead (Φ = 2.0 mm) which have spherical shape were loaded on the quartz wool. In order to pack the plasma discharge gap, the height of catalyst bed exceeded 20.0 mm. K type thermocouple was utilized to measure and record the catalyst bed temperature. Because the thermocouple could function as a ground electrode and be affected by plasma, thermocouple was protected by another quartz tube and located just below the quartz wool though it might not be exact temperature of catalyst bed.

**Figure 6:**
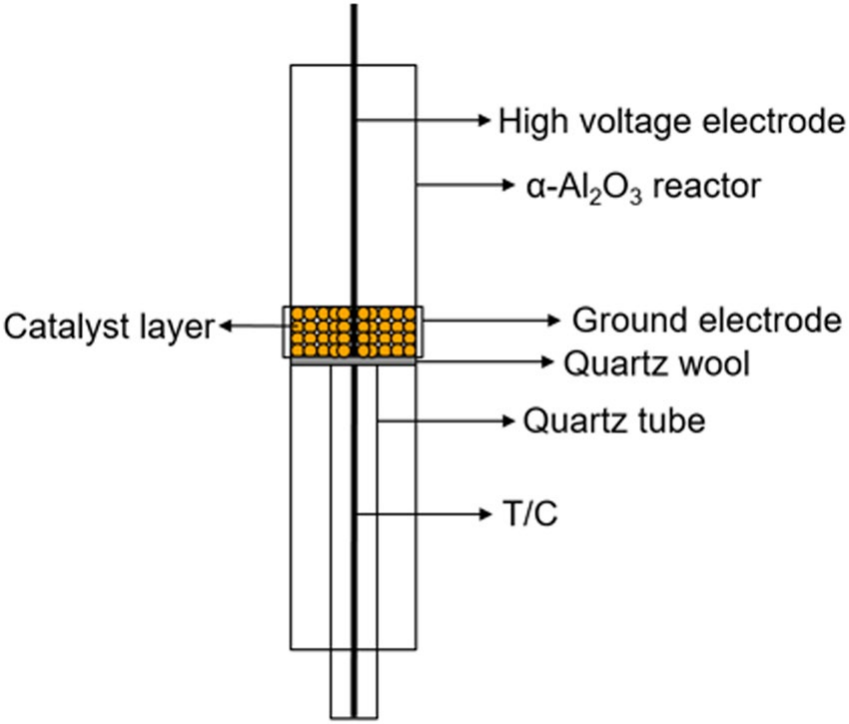
Shape of the plasma-catalyst hybrid reactor.

Shape of the plasma-catalyst hybrid reactor is shown in Fig. 6. The catalyst zone and the plasma zone were combined into one reaction region, which led to the overlap of them at the same position in this reactor. Thickness of the stainless steel rod that was located at the middle of reactor was 4.0 mm. Stainless steel plate that had a length of 20.0 mm and a thickness of 0.2 mm surrounded the outer surface of the reactor. Stainless steel rod acted as high voltage electrode and stainless steel plate did as ground electrode. Accordingly, the length of plasma zone was 20.0 mm, and the distance of discharge gap was 4.0 mm. As a result, the reaction volume was about 2.0 cm^3^ in plasma only condition. However, the packing material occupied the space where the plasma was generated in case of plasma-catalyst hybrid reaction, which led to the diminution of the plasma reaction volume. An arbitrary function generator (GW INSTEK AFG-2012) and high voltage amplifier (TREK 20/20C-HS) generated an AC high voltage with maximum of 8.5 kV_P-P_. This resulted in the creation of the plasma discharge. Except the input voltage, the experiments in this study were conducted under the same plasma conditions including sinusoidal waveform and driving frequency (4 kHz).

### Catalyst preparation

Commercial 2.0 mm borosilicate glass bead (Sigma Aldrich) was utilized as a support under DBD plasma condition. Then, various metal oxides were loaded on the spherical glass bead. The catalyst was prepared by following procedure^32^. The glass beads were etched with 5 M NaOH (≥98%, Sigma Aldrich) solution at 100 °C, followed by immersing it in a suspension of metal oxide. Next, the mixture was dried at 19 120 °C for 1 h and washed with distilled water. The operation was repeated several times until the glass beads were no longer transparent with desired content of metal oxide. MnO (99%, Sigma Aldrich), Mn_2_O_3_ (99.9%, Sigma Aldrich), MnO_2_ (≥99%, Sigma Aldrich), Fe_2_O_3_ (≥99%, Sigma Aldrich), NiO (99%, Sigma Aldrich), and Co_3_O_4_ (99.5%, Sigma Aldrich) were used as metal oxides. Fixed amount (1 wt%) of metal oxides were loaded to each of the catalysts. 4.0 g of catalysts were used in the reaction. X-Ray Diffraction (RIGAKU SMARTLAB) with a Cu Kα radiation operated at 40 kV and 50 mA was utilized to verify the solid-state phases of the catalysts. In addition, the N_2_ adsorption/desorption method by an ASAP 2010 instrument (Micromeritics Co.) was employed in order to obtain the specific surface area of the catalysts.

### Activity measurement

The plasma only reaction and the plasma-catalyst hybrid reaction were carried out at atmospheric pressure utilizing the same α-Al_2_O_3_ reactor. The reactants were 20% CH_4_, 10% O_2_, balanced in Ar. In addition to Ar, He can be used as balance gas or discharge gas. However, the Ar plasma is known to have better energy transfer efficiency compared to the He plasma under same conditions^33^. Thus, Ar selected as balance gas. The total gas flow was maintained at 100 ml/min. Mass flow controllers (SIERRA) controlled the flow of each gas. Gas chromatography (AGILENT GC 6890 N) equipped with a thermal conductivity detector (TCD) and a flame ionization detector (TCD) detected the product gases after the reactant gases went through the discharge region. The line from the latter part of the reactor to the gas chromatography was heated up to 120 °C, so all products were vaporized. The columns inside the GC were CP-7429 capillary column for TCD, and DB-5 capillary column for FID. CP-7429 column was composed of two parallel columns (CP-Molsieve 5 Å and PoraBOND Q) which allowed us to detect H_2_, O_2_, CO, CH_4_, CO_2_, C_2_H_2_, C_2_H_4_, C_2_H_6_, C_3_H_6_, and C_3_H_8_. By DB-5 column, CH_3_OH and HCHO were identified. Methane conversion and methanol yield were obtained at low temperature. However, the reaction temperature was changed from room temperature to about 100 °C depending on the plasma condition. Methane conversion, and selectivity and yield of CO, CO_2_, and CH_3_OH are acquired in the following:

1 $${\rm{Conversion}}\,(C{H}_{4})=\,\frac{mole(consumed\,C{H}_{4})}{mole(introduced\,C{H}_{4})}\times 100\,[ \% ]$$

2 $${\rm{Selectivity}}\,({\rm{C}}0)=\,\frac{mole(produced\,CO)}{mole(converted\,C{H}_{4})}\times 100\,[ \% ]$$

3 $${\rm{Selectivity}}\,(C{O}_{2})=\,\frac{mole(produced\,C{O}_{2})}{mole(converted\,C{H}_{4})}\times 100\,[ \% ]$$

4 $${\rm{Selectivity}}\,(C{H}_{3}OH)=\,\frac{mole(produced\,C{H}_{3}OH)}{mole(converted\,C{H}_{4})}\times 100\,[ \% ]$$

5 $${\rm{Yield}}\,({\rm{Product}})=\,\frac{Conversion(C{H}_{4})\times Selectivity(Product)}{100}\,[ \% ]$$
